# Multi-synaptic boutons are a feature of CA1 hippocampal connections in the *stratum oriens*

**DOI:** 10.1016/j.celrep.2023.112397

**Published:** 2023-04-18

**Authors:** Mark Rigby, Federico W. Grillo, Benjamin Compans, Guilherme Neves, Julia Gallinaro, Sophie Nashashibi, Sally Horton, Pedro M. Pereira Machado, Maria Alejandra Carbajal, Gema Vizcay-Barrena, Florian Levet, Jean-Baptiste Sibarita, Angus Kirkland, Roland A. Fleck, Claudia Clopath, Juan Burrone

**Affiliations:** 1 MRC Centre for Neurodevelopmental Disorders, Institute of Psychiatry, Psychology and Neuroscience, King’s College London, London SE1 1UL, UK; 2 Centre for Developmental Neurobiology, Institute of Psychiatry, Psychology and Neuroscience, King’s College London, London SE1 1UL, UK; 3 The Rosalind Franklin Institute, Harwell Campus, Didcot OX11 0FA, UK; 4 Bioengineering Department, Imperial College London, London, UK; 5 Centre for Ultrastructural Imaging (CUI), Kings College London, New Hunts House, Guys Hospital Campus, London SE1 1UL, UK; 6 University Bordeaux, CNRS, Interdisciplinary Institute for Neuroscience, IINS, UMR 5297, 33000 Bordeaux, France; 7 University Bordeaux, CNRS, INSERM, Bordeaux Imaging Center, BIC, UAR3420, US 4, 33000 Bordeaux, France

**Keywords:** multi-synaptic bouton, synapse, axon, dendrite, hippocampus, network synchrony, connectome

## Abstract

Excitatory synapses are typically described as single synaptic boutons (SSBs), where one presynaptic bouton contacts a single postsynaptic spine. Using serial section block-face scanning electron microscopy, we found that this textbook definition of the synapse does not fully apply to the CA1 region of the hippocampus. Roughly half of all excitatory synapses in the *stratum oriens* involved multi-synaptic boutons (MSBs), where a single presynaptic bouton containing multiple active zones contacted many postsynaptic spines (from 2 to 7) on the basal dendrites of different cells. The fraction of MSBs increased during development (from postnatal day 22 [P22] to P100) and decreased with distance from the soma. Curiously, synaptic properties such as active zone (AZ) or postsynaptic density (PSD) size exhibited less within-MSB variation when compared with neighboring SSBs, features that were confirmed by super-resolution light microscopy. Computer simulations suggest that these properties favor synchronous activity in CA1 networks.

## Highlights

• Half of all synapses onto CA1 basal dendrites are multi-synaptic boutons (MSBs)
• Each MSB can contact up to 7 dendritic spines from different cells
• Synapses made by the same MSB share similar morphological properties
• Simulations suggest MSBs promote network synchrony

## Graphical abstract

**Figure:**
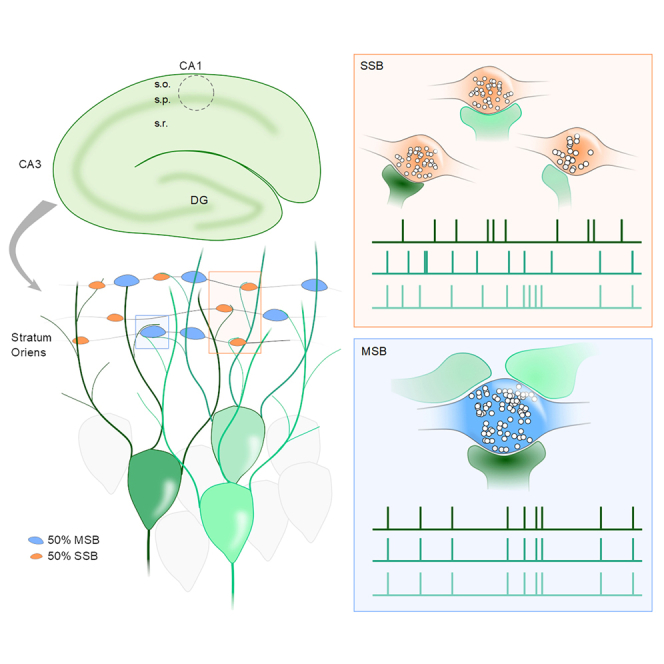

## Teaser

Ribgy et al. show that roughly half of all synapses formed onto hippocampal CA1 basal dendrites in *stratum oriens* are multi-synaptic boutons (MSBs): excitatory synapses formed by one presynaptic bouton onto multiple postsynaptic spines from distinct cells. Their large numbers and morphological properties suggest that they may help promote network synchrony.

## Introduction

In mammalian brains, chemical synapses are usually thought of as bicellular units formed by a single presynaptic bouton contacting a single postsynaptic compartment. These connections are typically referred to as single synaptic boutons (SSBs) and are widespread throughout the brain. However, a closer look at synaptic morphologies from electron microscopy studies show that alternative arrangements also exist.^1^ They include single postsynaptic compartments that receive multiple presynaptic inputs (multi-synaptic spines [MSSs]) and single presynaptic boutons that form synapses onto multiple postsynaptic sites (also known as multi-synaptic boutons [MSBs]).^2^ Even though these apparently rarer configurations represent an interesting alternative to the prototypical synapse, their distribution and prevalence in the brain, as well as their morphological properties and functional role in neuronal circuits, are not well established.

MSBs are particularly curious configurations that have been intimately associated with long-term forms of structural plasticity in the brain.^3^^,^^4^ For example, the number of MSBs in the cerebellar and motor cortices has been shown to increase following the emergence or modulation of complex motor skills^5^^,^^6^ and can be actively modulated in the visual cortex by either sensory deprivation^7^ or exposure to complex visual scenes.^8^ Similarly, associative learning paradigms (eye blink conditioning) in rabbits showed an increase in the fraction of MSBs in the *stratum radiatum* of the hippocampal CA1 subfield.^9^ However, the mechanisms responsible for the appearance of MSBs are less well understood. Early studies showing that estrogen increased the density of spines in the hippocampus also found that it resulted in an increase in the number of MSBs, suggesting that new spines may target existing boutons to create MSBs. Indeed, work in the cortex performing correlative *in vivo* imaging of spines together with electron microscopy (EM) reconstructions showed that newly formed spines were preferentially associated with MSBs,^10^ and spinogenesis induced by long-term potentiation (LTP)-like stimuli in hippocampal slices observed newly formed spines synapsing onto preexisting boutons.^11^^,^^12^ The presynaptic bouton of an MSB can therefore be seen as an anchor point for new spines to latch onto to form new synaptic connections. In this context, MSBs have been postulated to represent transient nodes of synaptic competition, where synapses compete to become the sole target of the presynaptic bouton.^10^^,^^13^ Functionally, however, the role played by MSBs in network activity is less clear. In fact, whereas some studies have proposed that they serve to synchronize network activity,^14^ others have observed that MSBs form weak synapses that will have little impact on the network.^13^ Behaviorally, in the monkey prefrontal cortex, the modulation of MSB number with estrogen was shown to correlate with measures of working memory, suggesting that MSBs may play some role in cognitive function. Together with the activity-dependent plasticity mechanisms described above, MSBs appear to be an important yet understudied neuronal structure linked to key physiological processes in the brain.

EM reconstructions have shown that although MSBs appear to be present in many brain areas, including the hippocampus and cortex, they are typically in the minority.^10^^,^^15^^,^^16^^,^^17^ Here, we show that for excitatory synapses in the *stratum oriens* (SO) of the hippocampal CA1 subfield, MSBs are present in roughly equal proportion to SSBs. What is more striking is that these MSBs involve single boutons that form contacts onto multiple spines from different dendrites of different cells. By looking at EM reconstructions of two different ages (postnatal day 22 [P22] and P100), we show that MSBs are not a developmental anomaly but instead represent a feature of how CA1 hippocampal neurons receive inputs. Moreover, the properties of synaptic contacts within single MSBs are less heterogeneous than those observed across neighboring SSBs, suggesting that MSBs convey similar information across its many contacts. Using mathematical models of network activity that incorporate these properties, we propose that MSBs in the hippocampus may synchronize network activity by using single boutons to broadcast information to multiple postsynaptic partners.

## Results

**Figure 1:**
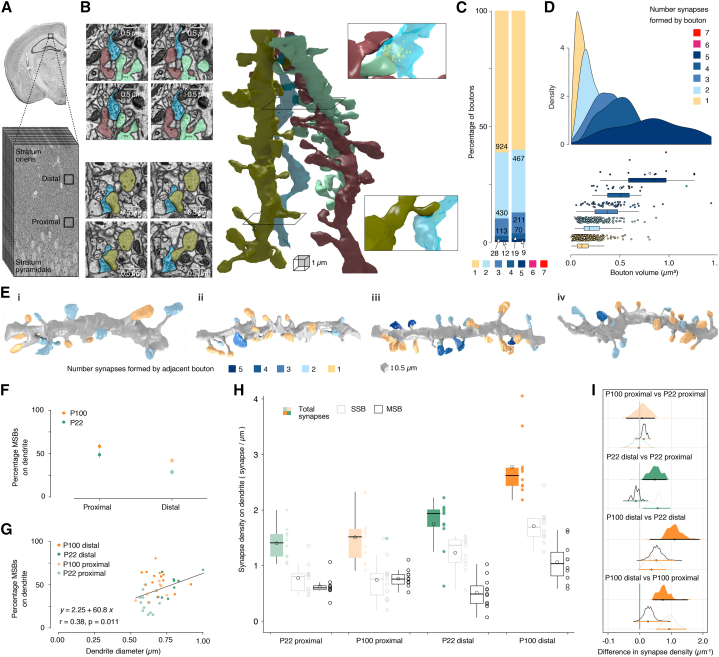
Serial block-face scanning electron microscopy reveals multi-synaptic boutons feature heavily in the *stratum oriens* (A) Low-magnification image of *stratum oriens* and *stratum pyramidale* of the hippocampus, where basal dendrites of CA1 pyramidal neurons reside. Black box represents the cubes of tissue extracted and imaged using serial block-face scanning EM. 2 cubes, 1 proximal to the *stratum pyramidale* and another more distally located from the CA1 somata region were processed from P22 and P100 mice. (B) 3D reconstructions of 3 dendrites (dark gray, olive green, and bright mint green) forming synapses onto a single axon (blue). Example images on the left and close-up 3D reconstructions on the right are the corresponding slices through the z stack. The top example shows a multi-synaptic bouton forming 2 connections; the bottom example shows a single synaptic bouton. (C) Boutons from both animals were classified depending on how many synaptic contacts they formed and plotted as a percentage of the total number of boutons. The left bar refers to boutons that form onto traced dendrites, whereas the right bar refers to boutons analyzed from selected axons. The color code represents the number of synapses formed by a bouton. (D) Density histogram of axonal bouton volume grouped by the number of contacts made. The greater the number of contacts formed, the greater the bouton volume (Krusal-Wallis p value: 1.82e−50, omega squared: 0.312). (E) 3D reconstructions of dendrites traced in the *stratum oriens* of 4 mice; P22 proximal (i), P22 distal (ii), P100 proximal (iii), and P100 distal (iv). Spine color indicates the number of contacts formed by the apposing bouton. Yellow refers to a spine forming onto a single synaptic bouton, blue spines represent multi-synaptic boutons, and the darker the shade of blue, the more synapses formed by the apposing multi-synaptic bouton. (F) Plot of the percentage of MSBs on either proximal or distal dendrites for both P22 (green) and P100 (orange) dendrites. (G) Plot of the percentage of MSBs as a function of dendrite diameter for all dendrites analyzed (P22, green; P100, orange) showing a correlation between the two. (H) Pooled data showing the density of total synapses formed on individual dendrites (filled), as well as the density of synapses broken down into those formed by single synaptic boutons (gray outline) and multi-synaptic boutons (black outline). Box-and-whisker plots indicate the median (black line), the 25th–75th percentiles (black box), and the 10th–90th percentiles (black whiskers); open circles within the box and to the right of the box represent the mean and individual values, respectively. (I) Age and region comparisons of synaptic densities visualized using estimation plots of effect sizes and their uncertainty using confidence intervals. Medians are indicated with a circle and the 95% confidence intervals with a horizontal line. Separation between the confidence interval and 0 was considered an effect. Confidence intervals were adjusted for multiple comparisons by an extension of the Ryan-Holm step-down Bonferroni procedure. 4 comparisons were made for total synaptic density, whereas confidence intervals were adjusted for 12 comparisons for synapse densities formed by single and multi-synaptic boutons.

We set out to look at the properties of synapses in the SO of the CA1 hippocampus, a region populated by basal dendrites, which receive a large fraction of the total number of inputs onto CA1 pyramidal neurons. We performed serial EM reconstructions of two regions of the SO, one proximal and another distal to the *stratum pyramidale* (Figure 1A), for both a young adolescent (P22) and an adult (P100) mouse brain. In each case, we reconstructed dendrites and axons within ∼1,000 μm^3^ volumes (cubes of roughly 10 × 10 × 10 μm), allowing us to establish the properties of synapses along the distance of basal dendrites to the soma, as well as across development. Having reconstructed hundreds of synapses, we noticed a salient feature in the properties of presynaptic terminals in the SO*.* Data pooled from all our reconstructions showed that nearly half (∼45%) of the boutons in this area formed synapses onto more than one postsynaptic spine, resulting in structures known as MSBs. More intriguingly, the spines contacted by MSBs belonged to different dendrites rather than to neighboring spines on the same dendrite (Figure 1B). From the general topology of dendritic arbors, we predicted that these dendrites likely belonged to different neurons, suggesting that MSBs were transmitting signals across different cells. To confirm this, we reconstructed neurons sparsely labeled with fluorescent proteins and mapped the 3D spatial arrangement of their dendrites (Figure S1). We found that dendrites tended to fan out from the soma along the SO (Figures S1A–S1H)*,* rarely coming into close contact with each other. A measure of potential contacts with dendrites from the same cell decreased with distance from the soma, becoming negligible beyond ∼50 μm (Figure S1I). Since the most proximal EM dataset was obtained at a distance of ∼50 μm (Figure 1A), dendrites from the same cell were unlikely to contribute more than a single spine to an MSB. In addition, we reconstructed MSBs from a larger tissue block (40 × 100 × 23 μm - in xyz) that included part of the *stratum pyramidale*, where we could identify four pyramidal cell bodies. By reconstructing a total of 1.4 mm of dendrite, including those from the four neurons with an identified soma, we found 13 MSBs where one of the dendrites could be assigned to a specific soma (Figure S1J–S1L), and the additional dendrite(s) could be traced for a minimum of 35 μm (mean: 75 μm; range: 35–146 μm; n = 13). We found that the dendrites of a given MSB appeared to originate from distinct cells—dendrites could be traced back to distinct locations in the *stratum pyramidale*, with non-overlapping somatic domains (see examples in Figures S1M–S1S). Together, our data strongly suggest that the vast majority, if not all, of the MSBs described here represent a single presynaptic bouton contacting the dendrites of multiple postsynaptic CA1 pyramidal neurons. Although a single MSB can contact up to 7 postsynaptic spines (Figure S2), the majority ranged from 2 to 5 contacts (Figures 1C, S2), with the proportion of MSBs decreasing progressively with the number of postsynaptic partners they contacted. Nevertheless, MSBs forming more than 3 contacts still represented as much as 15% of all boutons analyzed (Figure 1C). Interestingly, a given axon innervating the SO was capable of forming both MSBs and SSBs, suggesting that MSBs are unlikely to be a feature of a specific neuronal subtype. Morphologically, bouton volume scaled with the number of synapses formed, with boutons progressively increasing in size the more active zones they had (Figure 1D). We conclude that MSBs are relatively large structures that are abundant in the SO of the hippocampus and connect multiple CA1 neurons together.

Previous work has shown that the properties of synaptic boutons follow distance-dependent rules along the basal dendrites of CA1 pyramidal neurons.^18^ We therefore also explored the possibility that MSBs may follow a non-random spatial distribution. We found that the proportion of MSBs was highest in proximal dendrites for both P22 and P100 brains, reaching surprisingly high levels (>50%) in the proximal dendrites of the P100 brain (Figures 1E and 1F). Although the proportions remain high all along basal dendrites, distal dendrites showed a decrease in the fraction of MSBs, suggesting that there are distance-dependent rules that control the likelihood of finding an MSB in the SO (Figure 1F). Basal dendrites display a pronounced tapering with distance along the SO, and previous studies have used dendrite diameter as a proxy for measuring the distance of basal dendrite from the soma.^18^^,^^19^ We found that the proportion of MSBs also correlated with dendrite diameter, suggesting a graded decrease in the fraction of MSBs along dendrites, when moving away from the soma (Figure 1G).

A more intriguing picture emerged when looking at the absolute density of synapse types along dendrites. Distal dendrites showed an increase in overall spine density when compared with proximal dendrites, which was more apparent for older (P100) neurons and was mainly explained by an increase in distal SSBs (Figures 1H and 1I). In fact, there was a clear developmental increase in overall synapse density (for both SSBs and MSBs) from P22 to P100 in distal dendrites, suggesting that synapse formation continues beyond the third postnatal week in this area of the hippocampus (Figures 1H and 1I). Hierarchical clustering of dendritic domains based on SSB and MSB densities uncovered essentially 2 main groups populated by mostly proximal or distal domains, regardless of age (Figure S3). This distinction between proximal and distal dendritic domains is in line with previous findings showing distance-dependent differences in the synaptic properties of both postsynaptic spines and presynaptic boutons^18^^,^^19^ and strengthens the idea that different dendritic domains code for different types of information. Here, it appears that although MSBs are found in roughly equal numbers at all dendritic distances, the proportion of MSB synapses is biased toward proximal domains.

**Figure 2:**
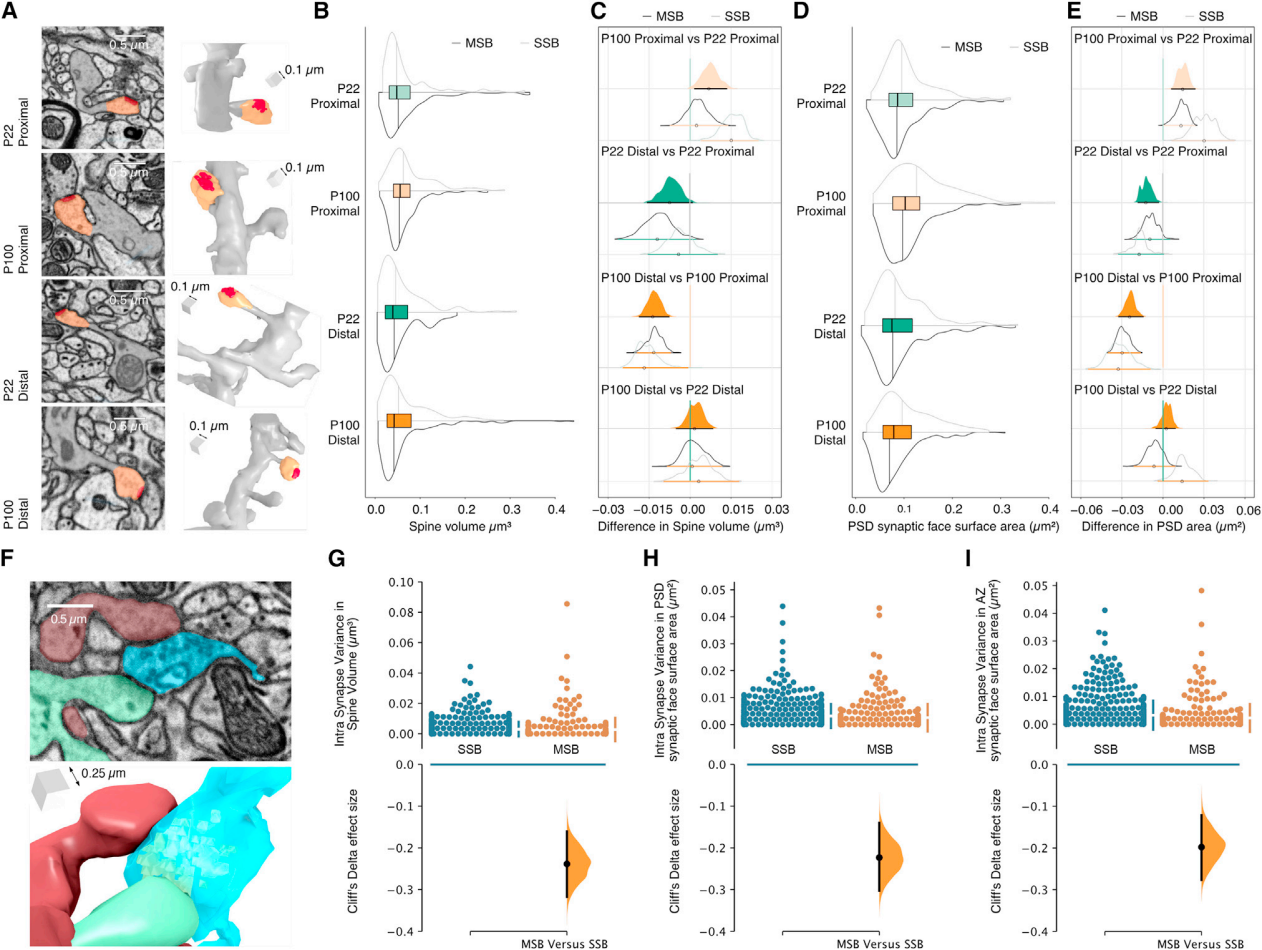
Properties of synapses formed by MSBs (A–E) Synapse strength increases from distal to proximal dendrites in the adult, but not juvenile, mouse. (A) Representative electron micrographs and corresponding 3D reconstructions of individual dendritic spines taken from mice of different ages and regions in the *stratum oriens*. (B) Combined plot describing the distributions of spine volumes for the total population (boxplot; as described above), those dendritic spines formed onto single synaptic boutons (gray split violin plot), and dendritic spines formed onto multi-synaptic boutons (black split violin plot). (C and D) Estimation plot of spine volume to visualize effect sizes and their uncertainty. Medians are indicated with a circle and the 95 confidence intervals with a horizontal line. Separation between the confidence interval and 0 was considered as an effect. Confidence intervals were adjusted for multiple comparisons by an extension of the Ryan-Holm step-down Bonferroni procedure. (D) Combined plot describing the distributions of PSD synaptic face surface areas for the total population (boxplot; as described above), those dendritic spines formed onto single synaptic boutons (gray split violin plot), and dendritic spines formed onto multi-synaptic boutons (black split violin plot). (E) Estimation plot of PSD synaptic face surface areas to visualize effect sizes and their uncertainty. Medians are indicated with a circle and the 95% confidence intervals with a horizontal line. Separation between the confidence interval and 0 was considered an effect. Confidence intervals were adjusted for multiple comparisons by an extension of the Ryan-Holm step-down Bonferroni procedure. (F–J) Synaptic properties exhibit greater homogeneity when from the same multi-synaptic bouton (MSB) than across individual single synaptic boutons (SSBs) on the same dendrite. (F) Representative electron micrograph and corresponding 3D reconstruction of MSB with 2 connecting spines. (G) Quantification of the variance in spine volume for neighboring SSBs (blue) and for spines formed onto single MSBs (orange). Vertical error bars indicate the 95% confidence interval (CI; two-sided permutation t test). Cliff’s Delta effect size between MSBs and SSBs variance is shown in the bottom panel. (H) Quantification of the variance in PSD size for spines on neighboring SSBs (blue) and for spines formed onto single MSBs (orange). Vertical error bars indicate the 95% CI (two-sided permutation t test). Cliff’s Delta effect size between MSBs and SSBs variance is shown in the bottom panel. (I) Quantification of the variance in AZ size for neighboring SSBs (blue) and for AZs within a single MSB (orange). Vertical error bars indicate the 95% CI (two-sided permutation t test). Cliff’s Delta effect size between MSBs and SSBs variance is shown in the bottom panel. Data from all mice are pooled together.

What are the properties of postsynaptic spines that connect to either MSBs or SSBs? Morphological measures of dendritic spines (Figure 2A) are intimately linked to their function: both spine volume and postsynaptic density (PSD) size correlate well with postsynaptic strength and have therefore regularly been used as structural measures of spine function.^20^^,^^21^ We found that at P22, spines contacting MSBs and SSBs were very similar in terms of volume and PSD size, whereas at P100, MSB spines were smaller (Figures 2B–2E), in line with previous work in the *stratum radiatum*.^13^ In general, morphological measures of postsynaptic spines increased in size from P22 to P100 for both MSBs and SSBs, particularly in proximal domains (Figures 2C and 2E). There was also an interesting spatial distribution of postsynaptic spine properties, where spines on both SSBs and MSBs showed an overall decrease in volume and PSD size with distance along a dendrite (Figures 2C and 2E), in agreement with previous observations.^18^^,^^19^ Together, our data show that spines formed onto MSBs are generally similar to SSBs at P22, but smaller at P100, and that dendritic location is an important determinant of spine size across all ages and synapse types.

**Figure 3:**
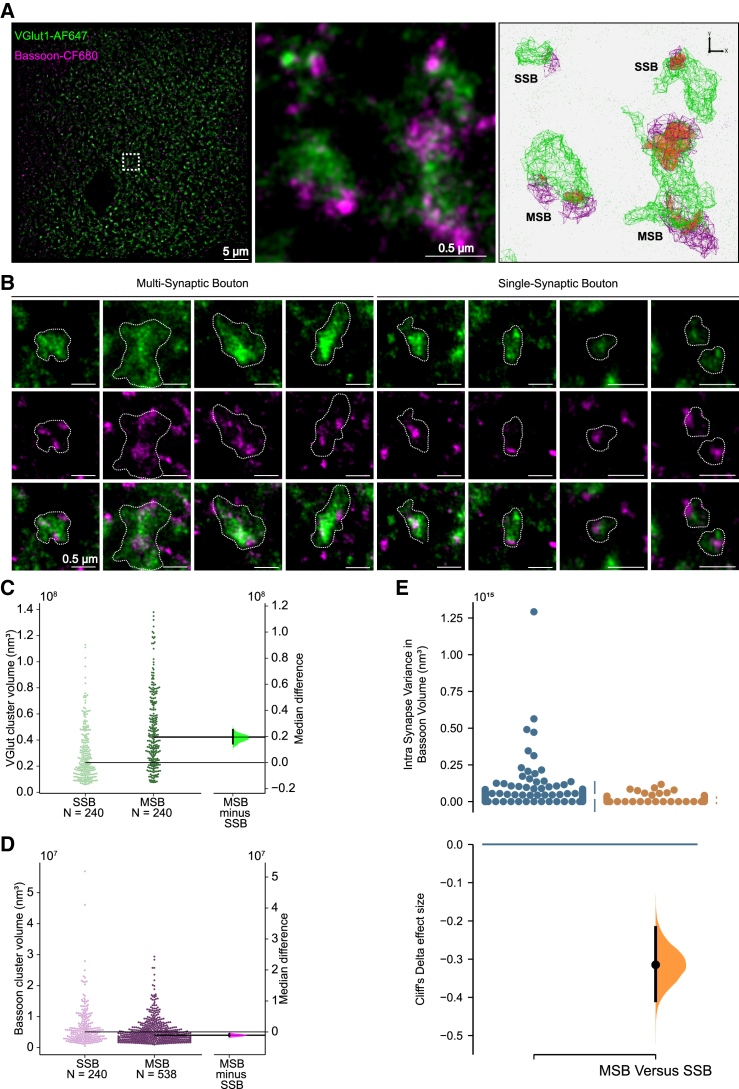
Super-resolution imaging reveals that AZ properties within a MSB are more similar than between SSBs (A) 2-color 3D dSTORM image of VGlut (green) and Bassoon (magenta) in the *stratum oriens* of a P22 mouse brain. Middle panel shows a zoomed-in view of four VGlut boutons (green) and their corresponding Bassoon-labeled AZ puncta (magenta). Right panel shows the clusters identified by the point cloud analyst (PoCA) approach used for automatic segmentation of vGlut and Bassoon, with the overlap between segmented clusters shown in orange. (B) Examples of MSBs and SSBs. (C) Quantification of VGlut cluster volume for SSBs and MSBs. Gardner-Altman estimation plot indicates the median difference in VGlut cluster volume between MSBs and SSBs. (D) Quantification of Bassoon cluster volume for SSBs and MSBs. Gardner-Altman estimation plot indicates the median difference in Bassoon cluster volume between MSBs and SSBs. (E) Quantification of the intrasynapse variance of Bassoon cluster volume for SSBs (blue) and MSBs (orange). Vertical error bars indicate the 95% CI (two-sided permutation t test). Cliff’s Delta effect size between MSBs and SSBs variance is shown in the bottom panel.

Synapses are highly heterogeneous entities, with both pre- and postsynaptic compartments showing large variations in structure and function.^22^^,^^23^ Although this heterogeneity is clearly present across both SSBs and MSBs, the variability in synaptic properties within single MSBs is less well understood. We therefore calculated the variance for active zone (AZ) size, PSD size, and spine volume of contacts within MSBs (for MSBs ranging from 2 to 5 contacts) and compared them with neighboring SSBs (from 2 to 5 neighbors) on a dendrite. Surprisingly, we found that all synaptic measures were more similar to each other within an MSB than across SSBs (Figures 2F–2I). To explore this further, we carried out dual-color direct stochastic optical reconstruction microscopy (dSTORM) of two presynaptic molecules, vGlut-1 and Bassoon, to uncover the 3D nanoscale arrangement of vesicles and their AZs, respectively, and to sample the properties of MSBs across multiple brains (Figure 3). Whereas staining for vGlut-1 showed clouds of vesicles that outlined presynaptic boutons, bassoon staining was predictably more punctate (Figures 3A and 3B). Once again, we found that a large proportion of boutons (∼25%) contained multiple AZs, ranging from 2 to 7. The lower number of MSBs identified with this method are likely due to a conservative approach to assigning AZ puncta to single vGlut clusters (Figure S4A), as well as to limitations in the resolution and imaging depth of 3D dSTORM in slices, which will strongly underestimate the number of AZs detected. In some cases, the locations of AZ puncta were clearly identified at opposite ends of a bouton, suggesting that postsynaptic partners were likely to be on different spines, as observed for MSBs using serial block-face scanning electron microscopy (SBFSEM). In line with the morphological characterization of bouton volume (Figure 1D) and AZ size, we found that MSBs had larger vGlut cluster volumes (Figure 3C) but no change in bassoon cluster volumes (Figure 3D) when compared with SSBs. More importantly, we found that bassoon cluster volume was less variable within MSBs than across SSBs (Figure 3E), in agreement with the decrease in variation measured for MSB AZ size. Together, our data suggest that MSBs transmit similar information at each of their contacts to multiple cells. Particularly, the similar AZ size is indicative of similar release probabilities, which would also imply similar short-term dynamics of neurotransmitter release.

**Figure 4:**
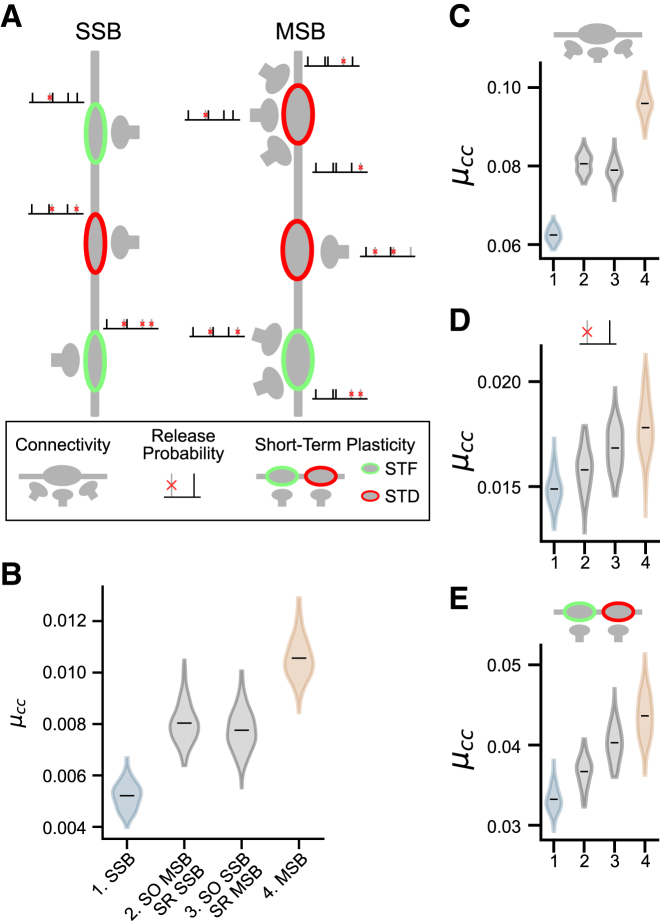
MSBs increase correlation in CA1 activity in computer simulations (A) Schematic of the simulation. Neurons from CA3 have multiple boutons that synapse onto neurons in CA1. Each bouton undergoes either short-term facilitation (STF; green boutons) or short-term depression (STD; red boutons). Each bouton has a fixed release probability. In the SSB simulations, each bouton connects to a single CA1 neuron, whereas in the MSB simulations, each bouton might connect to multiple neurons in CA1. (B) Mean correlation coefficient (μ_cc_) between neurons in CA1 for simulations run under four conditions: (1) where all inputs are SSBs, (2) where inputs to the *stratum oriens* (SO) include MSBs (50%) and those to the *stratum radiatum* (SR) are all SSBs, (3) where inputs to SO are all SSBs and those to SR include MSBs (25%), and (4) where all inputs include MSBs (50% for SO and 25% for SR). (C–E) Same as (B) but in setups with a single mechanism only: multiplicative connectivity (C), release probability (D), and STP (E). (B–E) Gray area shows distribution of μ_cc_ across 100 independent simulation runs.

In order to explore the functional role that MSBs could play in the hippocampus, we performed computer simulations in which CA1 neurons received synaptic input from CA3/2 neurons as well as background input. Inputs were divided into strata (*stratum radiatum* and SO), and the fraction of MSBs in each was set in accordance with either previously published data for the *stratum radiatum* (25% MSBs)^15^ or with the findings described here for the SO (50% MSBs). We set out to establish the impact on network activity of MSBs as a whole (across both strata) and in each stratum separately. We hypothesized that MSBs could influence the synchronization of CA1 pyramidal cells through three different mechanisms, namely a multiplicative effect (increased connectivity), release probability properties, and short-term plasticity (Figure 4A). We modeled the multiplicative effect by connecting each bouton from CA3/2 neurons to either a single neuron (SSB) or to multiple neurons (MSB) in CA1. To match the lower variance of AZ size within MSBs, we assumed that each bouton had a fixed release probability, drawn independently for each bouton, so that all AZs within a single MSB had an identical release probability (Pr). Furthermore, all synapses were subject to short-term plasticity, and each bouton was either facilitating or depressing, such that all AZs within a single MSB would have the same short-term plasticity (STP) properties. Our results showed that, for such a configuration, the correlation between activity of neurons in CA1 (measured as the correlation coefficient [$\mu_{cc}])$ is higher in networks with MSBs when compared with networks with SSBs only (Figure 4B). This was the case when MSBs were present in all strata, as well as when they were present only in single strata (*oriens* or *radiatum*). Importantly, MSBs in the SO were capable of increasing the correlated activity of the network on their own. This model implicitly assumes that the STP state of each bouton is independent of their release probability. However, Pr is known to correlate well with STP at classical synapses. We therefore matched the Pr of a bouton with an appropriate STP (high Pr boutons: depression; low Pr boutons: facilitation) and found similar results (Figure S4B).

Next, we wanted to check if a single mechanism alone was responsible for the increase in correlation or if all three mechanisms were contributing to the observed effect. We performed simulations in which only a single mechanism was active at a time and observed that all three mechanisms had small positive effects on the correlation of networks with MSBs (Figures 4C–4E). Together, our results show that MSBs could indeed play a role in synchronizing CA1 activity and suggest that multiple mechanisms could be involved in this synchronizing effect.

## Discussion

We show that a large proportion of synapses in the SO of the hippocampal CA1 subfield are MSBs rather than the more typical SSBs. Previous EM reconstructions have described MSBs in a number of brain regions, including the hippocampus and cortex. In the *stratum radiatum* of the hippocampus, where these structures were originally described, only 24% of all excitatory synapses were found to be MSBs, of which 20% formed connections onto different dendrites.^15^ Similarly, in the cortex, where a large number of synapses (∼1,700) have recently been annotated over a large 3D volume (0.3 mm^3^), 18% of all excitatory synapses were found to be MSBs,^16^ in agreement with previous work.^10^ Although MSBs are generally not considered to be a feature of neuronal circuits in the brain, it is gradually becoming apparent that the textbook definition of the synapse does not necessarily apply to all brain regions.^14^^,^^24^

In our study, we were unable to establish the identity of the axons that form MSBs, although many of the axons that innervate the basal dendrites of CA1 neurons originate from CA3 and CA2 pyramidal cells.^25^^,^^26^ A recent study in the *stratum lacunosum moleculare* showed that axons that form multiple clustered synapses onto the dendritic tufts of single cells, known as compound synapses,^27^ were also more likely to form MSBs, suggesting that, at least locally, there are some axonal properties that correlate with MSB formation. Importantly, they showed that compound synapses onto CA1 neurons were more likely to be formed by axons originating from entorhinal, rather than thalamic, neurons. However, entorhinal neurons projecting to the basolateral amygdala did not show a bias toward compound synapses, suggesting that connectivity rules depend on both the identity of the projection neuron and its target.^27^

The morphological properties of MSBs reveal much about the possible role they play in the brain. From a network perspective, the MSBs described here are intriguing in that they contact spines from different dendrites that belong to different neurons. As a result, they could act like synaptic transmission hubs, broadcasting signals across multiple CA1 neurons. Morphologically, MSBs in the SO are also unique in that the properties of synaptic contacts within an MSB are much less heterogeneous than between neighboring SSBs. As a consequence, MSBs are likely to transmit similar information across all AZs within a given bouton. Based on our mathematical models, we argue that these properties, when put together, all favor a role for MSBs in synchronizing activity across neurons in a network. CA1 neurons in the hippocampus show robust synchronous activity,^28^ particularly across neighboring cells,^29^ a feature that we suggest may emerge from the divergent connectivity pattern provided by MSBs. These findings, however, contrast with descriptions of MSB properties in the *stratum radiatum*^13^^,^^15^ and *lacunosum moleculare*,^27^ where spines on a given MSB were found to be more heterogeneous. In addition, in the *stratum radiatum*, serial-section EM and immuno-gold labeling of glutamate receptors in adult tissue showed that MSBs formed synapses with spines containing few glutamate receptors, suggesting that they were unlikely to result in substantial postsynaptic depolarization and were therefore also unlikely to participate in network synchrony.^13^ In contrast, a study in the dentate gyrus in young adults, where a surprisingly large fraction of synapses were classified as MSBs (∼72%), proposed that they likely played a role in driving synchronous network activity.^14^ It appears that the morphological properties of MSBs vary with age and/or brain area and are therefore also likely to impact on the role they play in network function.

Besides a possible role in synchrony, MSBs may also play an equally intriguing role in structural forms of synaptic plasticity.^10^ Work in the cortex has proposed that MSBs may represent transient entities that act as sites of synaptic competition, where postsynaptic spines that a share a single bouton compete for its dominance.^10^^,^^13^ *In vitro* imaging has previously shown that spines formed onto MSBs are indeed more dynamic than those onto SSBs,^30^ perhaps reflecting this competition. In this context, it is particularly interesting that *in vivo* measurements of dendritic spine turnover in the SO of the hippocampus is particularly high when compared with other brain regions such as the cortex,^31^^,^^32^ where the fraction of MSBs is low. Since spine turnover has been linked to the process of learning and memory,^33^^,^^34^^,^^35^ it is tempting to speculate that the higher density of MSBs in the hippocampus provides the necessary substrate for this plasticity to take place. In essence, MSBs would act as the docking sites for spines to latch onto as they appear, disappear, and reemerge during the acquisition, loss, or retrieval of memories.^10^ In addition to this role, MSBs may also be well positioned to explain a peculiarity of synaptic plasticity in the hippocampus—the spread of LTP to neighboring neurons.^36^^,^^37^ As previously proposed in an elegant review,^3^ the MSB could act as a node that spreads the potentiation of a given synaptic contact to all other synapses associated with it through some presynaptic mechanism. The lower variance of synapses associated with a single MSB described here further supports this notion.

### Limitations of the study

Aside from the lack of information on the identity of MSB axons (discussed above), one of the main limitations of the structural descriptions of synapses provided here is the lack of any functional correlate. Although we exploited the tight correlation between synapse structure and function for SSBs, which we used in our mathematical model, it is unclear how well these extend to MSBs. More work on the function of these intriguing structures is needed to properly assess their impact on neuronal circuits.

## STAR★Methods

### Key resources table

**Table:** 

REAGENT or RESOURCE	SOURCE	IDENTIFIER
**Antibodies**		

Rabbit Anti-vGlut1	Synaptic Systems	Cat#135 303; RRID:AB_887875
Mouse Anti-Bassoon	AbCam	Cat# ab82958; RRID:AB_1860018
Goat Anti-Rabbit IgG (H + L) conjugated to CF680	Biotium	Cat#20818
Goat anti-Mouse IgG (H + L) conjugated to Alexa 647	Invitrogen	Cat#A32728

**Deposited data**		

P22 mouse somatosensory cortex - Proximal	This paper	https://wklink.org/1289
P22 mouse somatosensory cortex - Distal	This paper	https://wklink.org/5367
P100 mouse somatosensory cortex - Proximal	This paper	https://wklink.org/1290
P100 mouse somatosensory cortex - Distal	This paper	https://wklink.org/1928
Computational model	This paper	https://doi.org/10.5281/zenodo.7685901

**Experimental models: Organisms/strains**		

C57BL/6 Mice	Charles River	C57BL/6
CD-1 Mice	In house	CD-1

**Software and algorithms**		

TrackEM2	Cardona et al., 2012^38^	https://imagej.net/TrakEM2
Neuromorph	Jorstad et al., 2015^39^	https://neuromorph.epfl.ch/index.html
NEST 2.20.0	Fardet et al., 2020^40^	https://zenodo.org/record/3605514#.Y_46qnbP1dg
ImageJ	NIH	https://mirror.imagej.net/
MATLAB	Mathworks	https://www.mathworks.com/products/matlab.html

### Resource availability

#### Lead contact

Further information and requests for resources and reagents should be directed to and will be fulfilled by the lead contact, Juan Burrone (juan.burrone@kcl.ac.uk).

#### Materials availability

This study did not generate new unique reagents.

### Experimental model and subject details

All animal procedures were approved by the local ethics committee and licensed under the UK Animals (Scientific Procedures) Act of 1986. Male and Female CD1 and C57BL/6 Mice were housed grouped in standard cages and provided with ad libitum food and water.

### Method details

#### Confocal imaging and dendrite tracing

Confocal imaging was performed using a Nikon A1 confocal microscope, equipped with a 40X water immersion objective (Olympus, Apo 40× WI λs DIC N3), and using NIS – Elements software. Images from 6 CA1 pyramidal cells sparsely labeled with Green Fluorescent Protein were obtained using high sampling resolution (pixel size 0.1 μm in X-Y and 0.3 μm or 0.8 μm in Z) and covering a large area of the basal dendritic tree (average area XY = 45551 μm2, average span Z = 45 μm).

Tracing was done using the Fiji Plugin Simple Neurite Tracer^41^ and traces were smoothed and re-sampled at constant node inter-distance of 0.5 μm using MATLAB TREES Toolbox. Distances to the soma for each node were calculated using the Pvec_tree function of MATLAB TREES.^42^ Custom written functions in IgorPro 6.37 were written to calculate 3D Euclidean distances between each node and all the other nodes located in separate branches from the same dendritic tree. A threshold of 5 μm was used to determine the number of separate branches judged to be at a close enough distance to each node to contribute spines to multi-synaptic boutons. Nodes were ordered by distance to cell soma and average values across 10 distance bins comprising the same number of nodes is shown for each cell. A total distance of 8179 μm of dendrites was traced across 6 cells (average 1363 ± 158 μm per cell).

#### Electron microscopy

Two C57BL/6 male mice (postnatal day 22 and 100) were transcardially perfused with 20 mL of ice-cold saline solution followed by 200 mL of ice-cold fixative (2% PFA and 0.2% glutaraldehyde mixture in 0.1 M phosphate buffer), followed by incubation overnight in fresh fixative at 4°C. Coronal vibratome sections (60 mm) were cut using a Leica VT1000S vibratome and further fixed in 1.5% potassium ferrocyanide: 2% osmium tetroxide in cacodylate buffer for 30 min at 4°C. Tissue was then thoroughly rinsed in distilled water and incubated in 1% aqueous thiocarbohydrazide for 4 min. After further rinsing, the samples were treated with 2% aqueous osmium tetroxide for 30 min, rinsed and en-bloc stained in 1% uranyl acetate for 2 h. To further enhance contrasts in the samples, one last treatment with Walton’s Lead was carried out for 30 min at 60°C, before proceeding to dehydration in an ethanol series and infiltration with Durcupan ACM resin (Sigma). After embedding and curing, tissue blocks were mounted on Gatan 3View aluminum pins using conductive glue (CircuitWorks Conductive Epoxy) and trimmed accordingly. Before imaging, samples were gold coated to increase electron conductivity. The specimens were then placed inside a Jeol field emission scanning electron microscope (JSM-7100F) equipped with a 3View 2XP system (Gatan). Section thickness was set at 40 nm (Z resolution). Samples were imaged at 2.5kV under high vacuum using a 2048 × 2048 scan rate, which gave a final pixel size of 4.4 nm. A third mouse was transcardially perfused and the brains processed as above. In this case, the region of the hippocampus covered a larger area of CA1 (total area 40 × 100 × 23.3 μm - xyz) and included both the stratum pyramidale (partially), as well as the *stratum oriens*. The specimen was placed inside a Jeol field emission scanning electron microscope (JSM-7800F) equipped with a 3View 2XP system (Gatan). Section thickness in this case was set at 50 nm (Z resolution) and sampled to achieve a final pixel size of 5.005 nm. This dataset was used in Figure S1J–S.

Electron microscope images were registered and manually segmented using the ImageJ plugin TrakEM2.^38^ Extracted 3D structures were exported to the Blender software with the Neuromorph toolset,^39^ which was used to compute surface, volume and length measurements and render 3D reconstructions.

#### Reconstruction of neurites

Stacks of SBFSEM images were concatenated and aligned using TrackEM plugin within the ImageJ environment.^43^ Portions of dendrites from proximal and distal regions of the stratum oriens were manually traced also using the TrackEM plugin. Only dendrites that could be followed for more than 5 μm through the stack were traced. When a dendritic spine was formed onto a bouton, that bouton was traced, as well as any other dendritic spines that synapsed upon the same bouton.

Extracted 3D structures were exported to the Blender software using the Neuromorph toolset to render 3D reconstructions^39^ where surface area, volume, and length measurements were extracted. The P22 dataset includes a subset of synapses included in a previous publication,^18^ which was expanded for this study. For tracing of dendrites on the larger area dataset (used for Figures S1J–S1S), five somas were identified (4 neurons and one putative astrocyte) by their nucleus. All dendrites that could be followed to the neuronal somas were traced throughout the imaged area. Multisynaptic boutons synapsing onto these dendrites were traced, as well as any other dendrites that could be followed for over 30 μm.

#### Histology for dSTORM

Mice (CD1, 22 days old) were anesthetized with an overdose of sodium pentobarbital and transcardially perfused with 30 mL of ice-cold saline solution followed by 40 mL of 4% (w/v) PFA (pH 7.3). The brains were carefully removed and put in 4% (w/v) PFA solution overnight at 4°C. Brains were incubated in 15% sucrose then in 30% sucrose, both overnight at 4°C. They were embedded in blocks of Gelatine/Sucrose and froze in isopentane (−60°C) and then stored at −80°C until cryo-sectioning. Coronal slice of 40μm thick were obtained with a cryostat (Leica). Slices were kept in anti-freeze PBS at −20°C. On the day of staining, slices were incubated with warm PBS 4 times 5 min each, to remove the gelatine/sucrose blocks. A 50mM NH4Cl solution was used to quench the remaining PFA. An antigen retrieval step was performed by incubating brain slices in Citrate buffer (10mM sodium citrate, 0.05% Tween 20, pH6) at 85°C for 25 min, followed by 3 PBS washes. For permeabilization, sections were incubated 4 times in a 0.25% Triton X-100 solution for 15 min each at room temperature (RT) on a shaker. Brain slices were incubated in a Blocking Buffer (5% BSA, 0.25% Triton X-100, 10% Goat Serum) for 2 h at RT on a shaker. This was followed by an overnight incubation with primary antibodies diluted in an Antibody Buffer (1% BSA, 0.25% Triston X-100, 5% Goat serum). On the following morning, slices underwent four 15 min PBS-Triton X-100 (0.1%) washes, followed by a 2-h incubation at room temperature with secondary antibodies diluted in the Antibody Buffer. The secondary antibody solution was washed off as before and slices were mounted onto 1.5H 25mm glass coverslip (Marienfeld). Slices were allowed to dry on the coverslips, melted 2% agarose was used to immobilize the tissue, and coverslip were kept in PBS at 4°C until imaging.

VGlut was labeled using an anti-VGlut1 (Rabbit, Synaptic Systems – 135 303) diluted at 1/500 and an anti-Rabbit coupled to CF680 (Goat, Biotium – 20818) diluted at 1/1000.

Bassoon was labeled using an anti-Bassoon (Mouse, Abcam – ab82958) diluted at 1/500 and an anti-Mouse coupled to AF647 (Goat, Invitrogen – A32728) diluted at 1/1000.

#### Multicolor 3D-dSTORM imaging

For dSTORM, coverslips were placed in an imaging chamber (AttoFluor Cell Chamber, Invitrogen). The imaging chamber was filled with STORM Buffer and sealed using another glass coverslip. Final dSTORM imaging buffers were composed of oxygen scavengers (100 μg/mL Glucose oxidase (Sigma-Aldrich G2133) and 4 μg/mL Catalase (Sigma-Aldrich C100)) and reductive agent (100mM β-Mercaptoethylamine-HCl, Sigma-Aldrich M6500). Oxygen scavengers stock solution containing 20mM Tris–HCl pH 7.2, 4mM TCEP, 25mM KCl, 50% Glycerol and 1 mg/mL Glucose oxidase (Sigma-Aldrich G2133) and 42 μg/mL Catalase (Sigma-Aldrich C100) and was stored at −20°C. Reducing agent stock solution containing 1M β-Mercaptoethylamine-HCl (Sigma-Aldrich M6500) in deionized water and pH adjusted at pH8 with NaOH, was stored at −20°C. Dilution STORM Buffer containing 100 mg/mL Glucose (Sigma-Aldrich) and 10% Glycerol (Fisher Scientific) in deionized water, was stored at 4°C.

Multicolor dSTORM imaging was performed using a spectral-demixing SAFeRedSTORM module (Abbelight, France) mounted on an Olympus IX3 equipped with an oil-immersion objective (100 × 1.5NA oil immersion, Olympus). The two fluorophores, AF647 and CF680 were excited with a single wavelength using a fibber-coupled 642nm laser (450mW Errol, France). Because the two fluorophores have a small shift in their emission spectrum, a long-pass dichroic beam splitter (700nm; Chroma Technology) was used to split the emission light on two cameras (ORCA-fusion sCMOS camera (Hamamatsu)). A photon ratio was calculated for each detection to assign the detection to one of the fluorophores.^44^^,^^45^ 3D-dSTORM imaging was performed by using cylindrical lenses placed before each camera.

Image acquisition and control of microscope hardware were driven by Abbelight’s *NEO* software. Region of interest (ROI) of 512 x 512 pixels (pixel size = 97nm) within the stratum oriens were identified. Depth of imaging was limited to the first ten micrometres of the slice due to the working distance of the objective and to allow a proper focus stabilization using the Olympus’s ZDC system. Image stacks contained 60,000 frames. Cross-correlation was used to correct for lateral drifts. N 3D calibration was performed using fluorescent beads (tetraspeck beads, invitrogen) to measure the x,y deformation obtained with the 2 cylindrical lenses on both cameras. With this information we calculated the z position of individual localizations with a precision of ∼1μm. Together with a Gaussian fit to single emitter events in the x-y plane (Maximum Likelihood Estimation, MLE, using the *NEO*_analysis software) we were able to obtain a the 3D spatial coordinates of single molecules with high resolution. A first transformation (cross-correlation) was applied to precisely realign detection events from both cameras. Finally, for each detection, a ratio of intensity between each camera was calculated (I1/(I1+I2)) in order to reassign the detection to one of the two fluorophores. Super-resolution images (.tiff) with a pixel size of 10nm and localization files (.csv) containing the 3D spatial coordinates of each demixed detection (detection of each fluorophores) were generated.

#### Multicolor 3D-dSTORM analysis

Point Clouds Analyst (PoCA),^46^ a continuation of SR-Tesseler^47^ and Coloc-Tesseler,^48^ was used to identify Single Synaptic and Multi Synaptic Boutons and to quantify VGlut(color 1) and Bassoon (color 2) clustering from localized molecule 3D coordinates. For each color, a 3D Voronoi diagram was computed by creating polyhedrons of various sizes centered on the localized molecules. Automatic segmentation allowed detecting clusters for each protein (VGlut cluster to identify excitatory presynaptic boutons, and Bassoon clusters to identify Active Zones) by using a density threshold^2^ for each color (δi1 and δi2, respectively) based on the mean density of molecules for the whole dataset, such that δi1 ≥ 4δd1 and δi2 ≥ 2δd2 for color 1 (VGlut) and color 2 (Bassoon), respectively. We only selected clusters with a minimum number of localizations, set to 200 and 50 localizations for color 1 and color 2, respectively. The colocalization between VGlut and Bassoon was computed from the overlapping clusters. Because of the stringent segmentation performed on color 1 (VGlut), which was needed due to the high density of synapses in brain slices, a significant amount of color 2 clusters (Bassoon) did not colocalize with color 1 clusters (Figure S3). To avoid colocalization issues, we manually picked 480 synapses of those identified automatically, where there was no sign of miscolocalization (see Figure S3), half of which were MSBs. Synapses were picked on multiples images from different brain slices from 3 mice. From these synapses, we extracted cluster volumes and localization densities for each color. Variances in cluster volume and density of multiple Bassoon clusters colocalizing with a single VGlut cluster (MSB) were measured and compared to randomized single Bassoon clusters which colocalized with a single VGlut cluster (SSB).

#### Computational model

All simulations were performed using the neural network simulator NEST 2.20.0^40^ and the code will be released on GitHub after publication.

#### Neuron model

Neurons from CA1 are current based leaky integrate-and-fire (LIF) with exponential postsynaptic currents. The sub-threshold membrane potential V of a neuron obeys the following equation:

(Equation 1) $$\tau_{m}\frac{dV}{dt}=-V+R\left(I_{b}+I_{syn}\right)\text{,}$$

where $\tau_{m}$ = 60 ms is the membrane time constant and R = 1GOhm is the input resistance. The background input $I_{b}$ is modeled as a Gaussian white noise current with mean $\mu_{b}$ = 12 pA and standard deviation $\sigma_{b}$ = 20 pA. The input current from presynaptic neurons $I_{syn}$ is modeled as the sum of input currents from all presynaptic neurons. Every time the membrane potential reaches a threshold value $V_{th}$ = 20mV, the neuron emits a spike. Following a spike, the membrane potential is reset to $V_{reset}$ = 10mV, and remains there for a refractory period $t_{ref}$ = 2 ms. In simulations without short-term plasticity (STP), the input current from a presynaptic neuron consists of their spike train filtered with an exponential with maximum amplitude A = 200 pA and time constant $\tau_{syn}$ = 1.5 ms. Neurons from CA3/2 are modeled as independent spike trains with Poisson statistics and rate r = 10 Hz.

#### Short-term plasticity

Where synapses are plastic, we use the STP model implemented on NEST 2.20.0,^40^ according to.^49^ The total synaptic input to a postsynaptic neuron *i* is given by:

(Equation 2) $$I_{syn}\left(i\right)=\sum_{j}Ay_{ij}\left(t\right)$$

where A is the absolute synaptic weight, and $y_{ij}$ determines the effective contribution of the postsynaptic current (PSC) from neuron *j* to the input current to neuron *i*. It evolves according to the system of equations:

$$\frac{dx}{dt}=\frac{z}{\tau_{rec}}-ux\delta \left(t-t_{sp}\right)$$

$$\frac{dy}{dt}=\frac{-y}{\tau_{syn}}+ux\delta \left(t-t_{sp}\right)$$

(Equation 3) $$\frac{dz}{dt}=\frac{y}{\tau_{syn}}-\frac{z}{\tau_{rec}}$$

where x, y and z are the fraction of synaptic resources in the recovered, active and inactive states respectively, $t_{sp}$ denotes the timing of a presynaptic spike, $\tau_{syn}$ is the decay time constant of PSCs and $\tau_{rec}$ is the recovery time constant for depression. The variable u describes the effective use of synaptic resources by each presynaptic spike, and it evolves according to:

(Equation 4) $$\frac{du}{dt}=\frac{-u}{\tau_{fac}}+U\left(1-u\right)\delta \left(t-t_{sp}\right)$$

where $\tau_{fac}$ is the time constant for facilitation. The parameters used were extracted from.^50^ For synapses undergoing facilitation, A = 1540 pA, U = 0.03, $\tau_{rec}$ = 130 ms, $\tau_{fac}$ = 530 ms. For synapses undergoing depression, A = 250 pA, U = 0.5, $\tau_{rec}$ = 800 ms, $\tau_{fac}$ = 0 ms.

#### Correlation coefficient

We calculate the Pearson’s correlation coefficient between spike trains of every pair of postsynaptic neurons. All simulations are run for 51 s. Spike trains are created from the last 50 s of simulation, with bins of 20 ms. Each independent simulation run generates a single mean value of correlation coefficient $\mu_{cc}$, across all pairs of postsynaptic neurons. Figures show distribution of $\mu_{cc}$ across 100 independent simulation runs.

#### Full simulation

We simulate two networks representing CA1 and CA3/2, with 100 excitatory neurons each. CA3/2 neurons are modeled as independent spike trains with Poisson statistics and rate r = 10 Hz CA1 LIF neurons receive a background input $I_{b}$ and synaptic inputs $I_{syn}$ from CA3/2 neurons. Synaptic inputs are divided into strata and can be either to *stratum radiatum* (SR, $I_{SR}$) or to *stratum oriens* (SO, $I_{SO}$). The total synaptic input to each neuron in CA1 is given by the sum of its inputs to SO and SR ($I_{syn}=I_{SO}+I_{SR}$). Each neuron from CA3/2 has 12 boutons, 5 of those connect to SO and the remaining 7 to SR. Each bouton may have one single or multiple active zones, as described in the following, and each active zone always connects to a single postsynaptic CA1 neuron. *Multiplicative*: when the connections to a given stratum are described as SSB, then each bouton has only one active zone which connects to one randomly chosen postsynaptic CA1 neuron. When the connections to a given stratum are described as MSB, then each bouton may have a number α of active zones, where α is randomly selected from the interval {α∈Z:1≤α≤5} with probabilities that depend on whether they connect to SO or SR. The number of active zones from the boutons that connect to SO are selected with probabilities extracted from Figure 1C. In this case, it follows from Figure 1C that the number of MSBs corresponds to approximately 45% of all synapses. The number of active zones from the boutons that connect to SR are selected with probabilities based on the distribution in Figure 1C, but adapted such that the number of MSBs corresponds to 25% of all synapses. Each active zone connects to one randomly chosen postsynaptic CA1 neuron. *Release probability*: Each bouton i has a release probability $p_{i}$, which is randomly chosen from a Gamma distribution with parameters shape k=2 and scale θ=0.15, and with values bounded to a maximum of 1 (Figure SA). The spike trains at different active zones within the same bouton i are different realizations of sampling from the same presynaptic spike train with the same probability $p_{i}$. *STP*: all connections are subject to STP. Each bouton is either undergoing short-term facilitation or short-term depression. The short-term plasticity state of each bouton applies to all active zones within that bouton.

#### Multiplicative only simulation

These simulations are the same as the full simulation, except that: (i) release probability $p_{i}=1$ for all boutons; and (ii) there is no STP.

#### Release probability only simulation

These simulations are the same as the full simulation, except that: (i) each presynaptic neuron from CA3/2 has either 5 boutons with a single active zone each (in the SSB scenario) or one bouton with 5 actives zones (in the MSB scenario). In both SSB and MSB scenarios, each active zone connects to one randomly chosen CA1 postsynaptic neuron; and (ii) there is no STP.

#### STP only simulation

These simulations are the same as the full simulation, except that: (i) each presynaptic neuron from CA3/2 has either 5 boutons with a single active zone each (in the SSB scenario) or one bouton with 5 actives zones (in the MSB scenario). In both SSB and MSB scenarios, each active zone connects to one randomly chosen CA1 postsynaptic neuron; and (ii) release probability $p_{i}=1$ for all boutons.

### Quantification and statistical analysis

Data analysis was performed, and graphical visualisation made, using R 3.4.1 (R Foundation for Statistical Computing; http://www.r-project.org/) and RStudio 1.2.5042 (https://rstudio.com).

Single variable data distributions were visualised using kernel density estimation plots. This non-parametric probability density function provided a more effective way to view the distribution of a variable.

Pooled synapse density and morphological measurements were visualised using violin or boxplots. Box-and-whisker plots indicate the median (thick horizontal line), the 25th–75th percentiles (box) and the 10th–90th percentiles (black whiskers); circles within and to the right of the box represent mean and individual values, respectively. Violin plots are comparable to boxplots, but give a representation of the kernel probability density of the data. Gray or black lines that extend from the center to the plot perimeter indicate the median of the separated MSB and SSB data. The box inside the violin represents the median and interquartile range of the combined MSB and SSB data.

Comparisons between synaptic densities and synapse morphologies were visualised graphically using estimation plots of effect sizes, and their uncertainty assessed using confidence intervals.^51^ This approach was preferred over traditional null hypothesis significance testing because of the associated limitations.^52^ Separation between the confidence interval and 0 was considered an effect. Confidence intervals were adjusted for multiple comparisons by an extension of the Ryan–Holm step-down Bonferroni procedure. Confidence intervals were adjusted for 4 comparisons for measures of synaptic density, whereas 12 comparisons were corrected for measures of synapse strengths. Differences between SSB and MSB synaptic strength variances were tested using Cliff’s delta and shown using Gardner-Altman estimation plots. 5000 bootstrap samples were taken; the confidence interval was bias-corrected and accelerated. For each permutation p value, 5000 reshuffles of the control and test labels were performed.

Hierarchical cluster analysis was performed with the ward minimum variance method using the nbclust package in R that aggregates 30 indices.^53^

Correlations were tested using Spearman’s rank-order correlation. P and r values are presented as equalities (two significant figures).

## Data Availability

• The serial block face scanning EM datasets used in Figures 1 and 2 have been deposited at WEBKNOSSOS (https://webknossos.org/) and are publicly available as of the date of publication. Accession numbers are listed here (https://wklink.org/1289; https://wklink.org/1290; https://wklink.org/5367; https://wklink.org/1928) and in the key resources table. • All original code has been deposited at https://github.com/juliavg/multi_synaptic_boutons and is publicly available as of the date of publication. DOI is listed in the key resources table. • Any additional information required to reanalyze the data reported in this paper is available from the lead contact upon request.

## Supplemental information

Document S1. Figures S1–S4

Video S1. Animation of 3D reconstruction of an MSB forming contacts with 7 spines on 7 different dendrites, related to Figures 1 and S2 The axon is shown in black, the MSB in yellow and the spines are shown in different colors.

Document S2. Article plus supplemental information
