# Supplementary material for: Multi-synaptic boutons are a feature of CA1 hippocampal connections in the stratum oriens
Source: Cell Rep. 2023 Apr 18;42(5):112397. doi: 10.1016/j.celrep.2023.112397 (PMC10695768; doi:10.1016/j.celrep.2023.112397)
Supplement: Document S1. Figures S1–S4 [file mmc1.pdf]

Cell Reports, Volume *42*

Supplemental information

Multi-synaptic boutons are a feature of CA1 hippocampal connections in the *stratum oriens*

Mark Rigby, Federico W. Grillo, Benjamin Compans, Guilherme Neves, Julia Gallinaro, Sophie Nashashibi, Sally Horton, Pedro M. Pereira Machado, Maria Alejandra Carbajal, Gema Vizcay-Barrena, Florian Levet, Jean-Baptiste Sibarita, Angus Kirkland, Roland A. Fleck, Claudia Clopath, and Juan Burrone

Figure S1

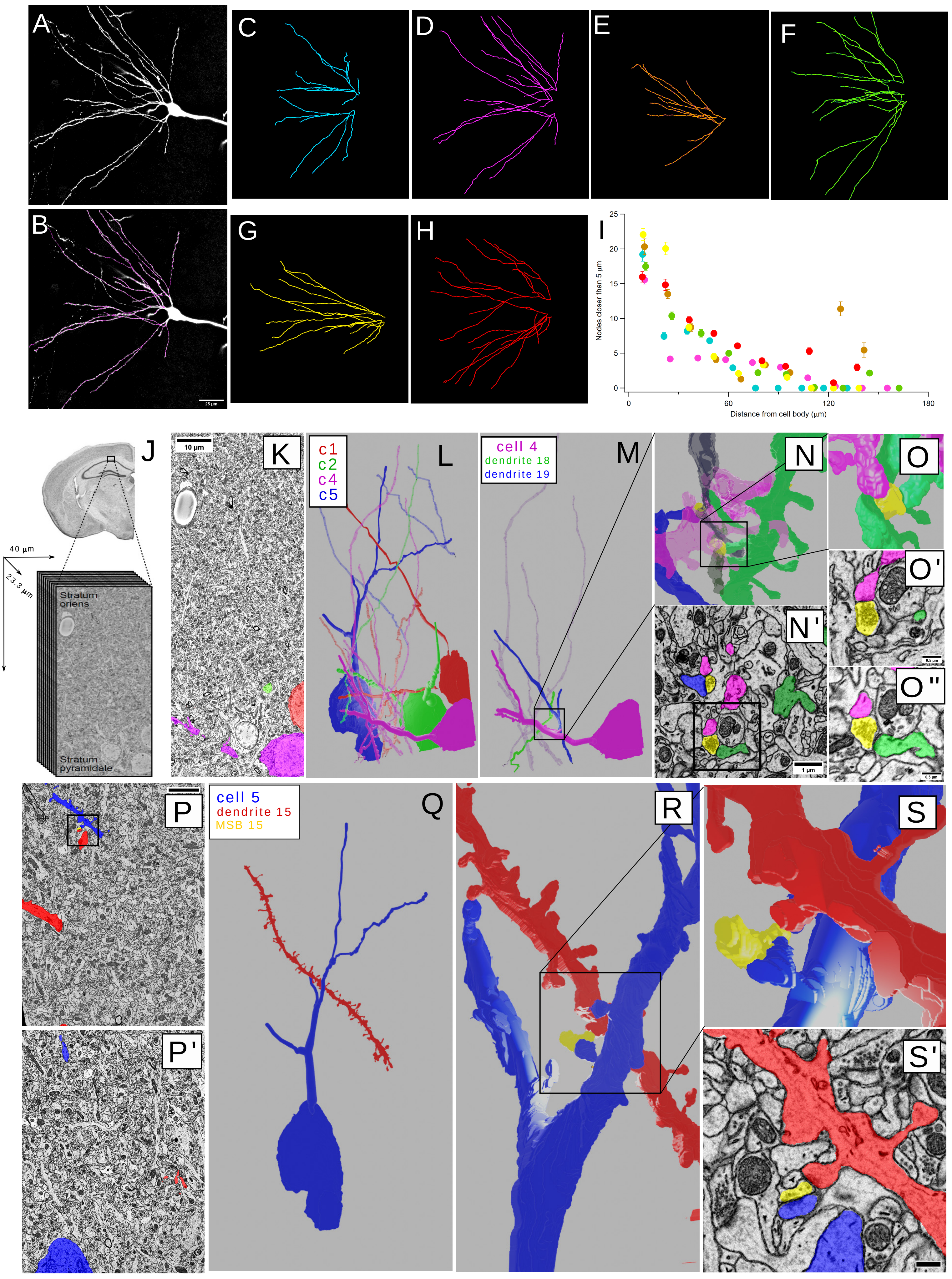
A
B
25 µm
C
D
E
F
G
H
I
Nodes closer than 5 µm
Distance from cell body (µm)
J
40 µm
23.3 µm
100 µm
Stratum oriens
Stratum pyramidale
K
10 µm
L
c1
c2
c4
c5
M
cell 4
dendrite 18
dendrite 19
N
N'
1 µm
O
O'
O''
0.5 µm
P
P'
Q
cell 5
dendrite 15
MSB 15
R
S
S'

Figure S1. The basal dendrites of a single CA1 hippocampal neuron are unlikely to contribute multiple synapses to MSBs, Related to Figure 1. (**A**) Example image of the basal dendrites of a CA1 pyramidal neuron transfected with EGFP using in utero electroporation. (**B**) Tracing of the basal dendrites of the neuron shown in A. (**C-H**) Maximal intensity projections of the tracings of the basal dendrites of 6 different CA1 pyramidal neurons used in this analysis. (**I**) A plot of the number of nearby dendrites with respect to a reference dendrite from the same cell, as function of distance from the soma. A 5 μm radius sphere (roughly 2 spine lengths) was moved along a reference dendrite and used as a way of assessing the proximity of dendrites from the same cell that could contribute to an MSB. A value of 1 indicates that a single other dendrite fell within the sphere and was therefore close enough to potentially contribute a spine to an MSB. The graph clearly shows that beyond 50 μm distance from the soma, the number of potential contacts is very low and cannot explain that large numbers of MSBs described here. (**J**) Low magnification image of an SBFSEM data set in the CA1 region of the hippocampus (obtained from a P22 mouse) that includes the region of the *stratum oriens* proximal to the *stratum pyramidale* and the distal edge of the *stratum pyramidale*. (**K**) Example EM image showing two cell bodies in *stratum pyramidale* (magenta and red) and a dendrite of another cell (green). (**L**) 3D reconstructions of 4 cell bodies and their dendrites (blue, red, magenta and green) together with 13 other non-assigned dendrites in the area that share an MSB with the soma-assigned dendrites. The non-assigned dendrites are in a lighter shade of the colour given to soma-assigned dendrites they share and MSB with. (**M**) The magenta soma and dendrites are shown together with the non-assigned dendrites they share and MSB with. Two of the non-assigned dendrites are highlighted in another colour (green and blue). Note how all dendrites appear to extend to regions distal to the magenta soma. (**N**) Zoomed-in reconstruction showing two MSBs formed by the magenta neuron: one shared with the blue dendrite and another with the green dendrite. Presynaptic terminal is shown in yellow and the axon in black. (**N'**) Single EM plane of both MSBs and dendrites shown in (N). (**O**) A higher zoom view of one the MSB formed with the green dendrite. Presynaptic terminal is shown in yellow. (**O'-O''**) Single plane EM images of MSB shown in (O). (**P-P'**) Single plane EM images of a soma-assigend dendrite (blue) and a non-assigned dendrite (red). (**Q**) Low zoom 3D reconstruction both dendrites, including the blue soma. (**R**) Zoomed-in 3D reconstruction of an MSB formed between the red and blue dendrites. Presynaptic terminal is shown in yellow. (**S**) A higher zoom view of the same MSB in (R). (**S'**) Single plane EM view of the MSB shown in (S). Scale bars (cubes): 10 μm in (**P**) and 0.5 μm in (**S'**).

Figure S2

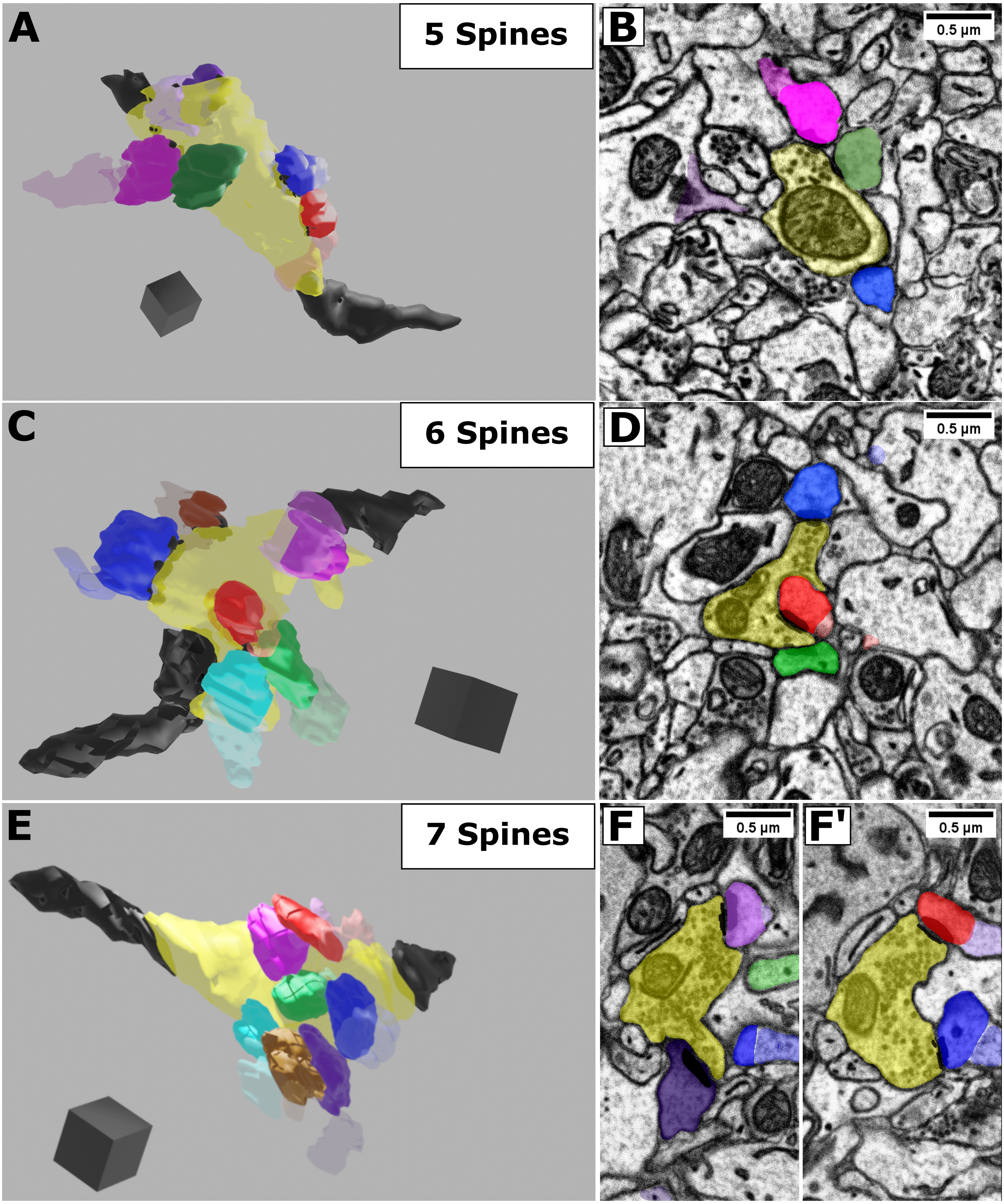

Figure S2. Example MSBs contacting multiple dendritic spines on different dendrites, Related to Figure 1. Reconstructions of MSBs contacting, from top to bottom, 5, 6 and 7 dendritic spines. The axon is shown in black, the MSB in yellow and the spines are shown in different colours. Each spine belongs to a different dendrites. Scale bar (cube): 250 nm.

Figure S3

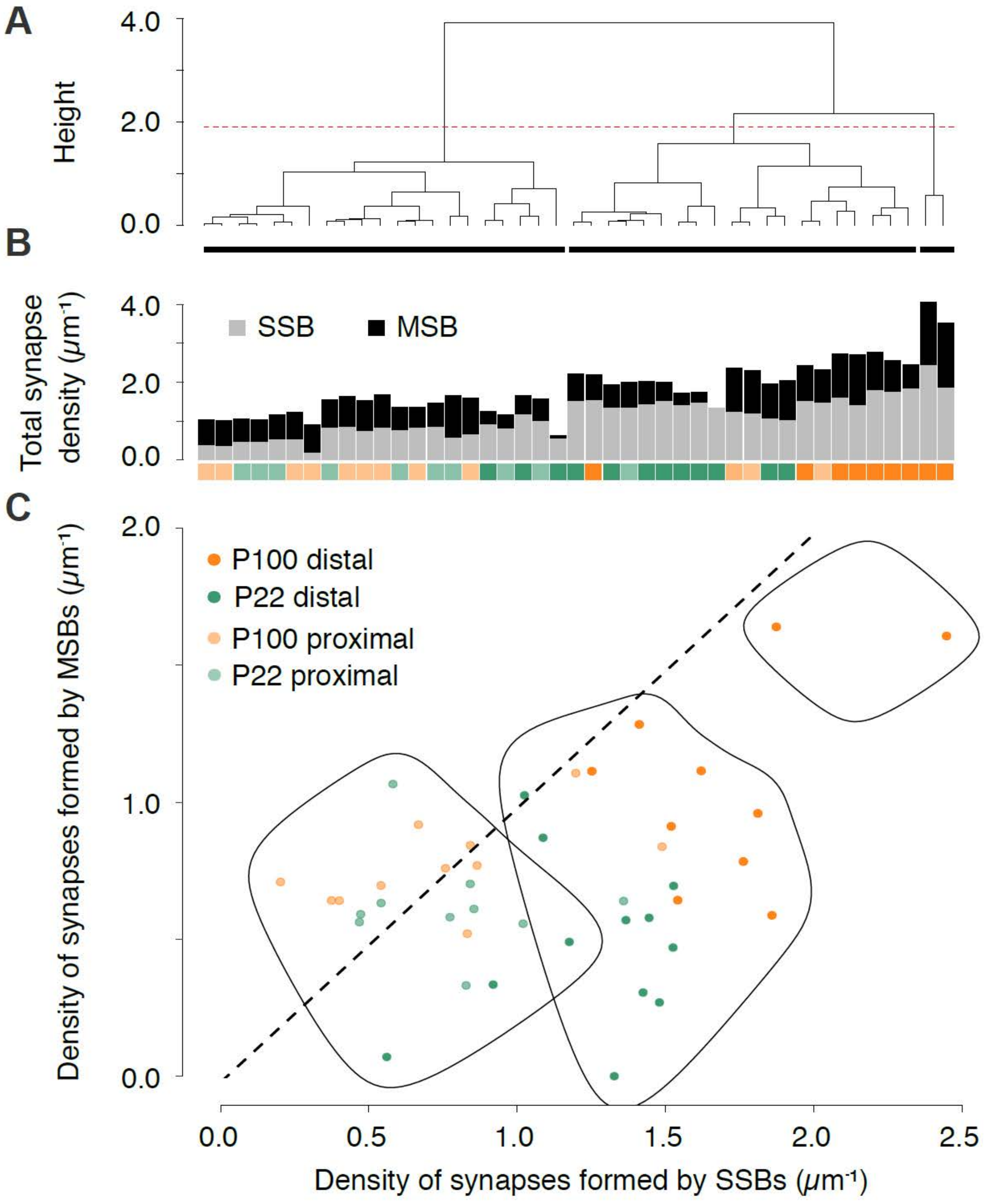

A
B
C
4.0
2.0
0.0
Height
Total synapse density (μm⁻¹)
SSB
MSB
Density of synapses formed by MSBs (μm⁻¹)
1.0
P100 distal
P22 distal
P100 proximal
P22 proximal
0.5
1.5
2.5
Density of synapses formed by SSBs (μm⁻¹)

Figure S3. Hierarchical clustering of synapses along basal dendrites show importance of synapse position, Related to Figure 1. (**A – C**) Hierarchical cluster analysis using the ward minimum variance method was used to determine if dendrites could be separated by their densities of single synaptic and multi synaptic boutons. By using the nbclust package in R that aggregates 30 indices (Charrad et al. 2012) the data was most appropriately categorised into 3 groups (red dotted line); dendrites that form roughly equal numbers of synapses onto MSBs and SSBs, dendrites that form more SSBs than MSBs and 2 dendrites which have a high density of both MSBs and SSBs. Without information on their region or age, dendrites quite accurately clustered according to whether they are distally or proximally located relative to CA1 somata, with distal dendrites forming equal contacts with MSBs and SSBs, whilst proximal dendrites tended to form spines onto SSBs.

Figure S4

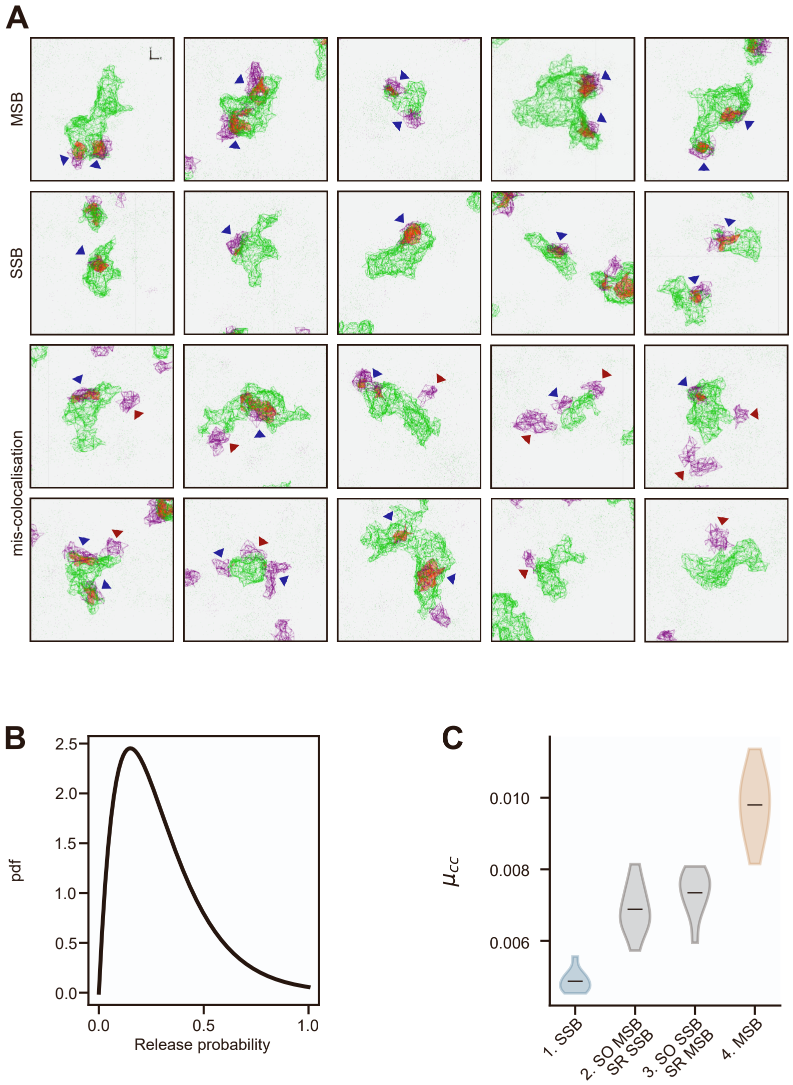

A
MSB
SSB
mis-colocalisation
B
pdf
2.5
2.0
1.5
1.0
0.5
0.0
0.0
0.5
1.0
Release probability
C
μ_{CC}
0.010
0.008
0.006
1. SSB
2. SO MSB SR SSB
3. SO SSB SR MSB
4. MSB

Figure S4. Limitations when imaging MSBs in brain slices and further modelling of the role of MSBs in network synchrony, Related to Figures 3 and 4. (**A**) Challenges associated with co-localisation of segmented clusters and their assignment into MSBs and SSBs. Multiple examples of identified MSBs (first row) and SSBs (second row), showing correct automated colocalisations (blue arrow heads). Third and fourth rows show putative colocalization errors from Bassoon puncta lying close, but outside a VGlut cluster (red arrow heads). This could result in an MSB being incorrectly assigned as an SSB (third row), in an MSB with an incorrect number of AZs (fourth row, examples 1 to 3), or in a bouton without any AZs (fourth row, examples 4 and 5). We took a conservative approach that only picked segmented clusters showing direct co-localisation (blue arrows). This approach will underestimate MSBs but avoids any false positive detections. (**B-C**) MSBs increase the correlation of CA1 neurons when the STP of a bouton is linked to its Pr. (**B**) Gamma distribution from which values of release probabilities are drawn. (**C**) Same as Figure 4B, but with non-independent release probability and short-term plasticity. The main model (Fig. 4) implicitly assumes that the short-term plasticity state of each bouton is independent of their release probability. As an alternative, here we simulate a scenario in which they are not independent. For each bouton, we draw a value of release probability p_i, which decides its short-term plasticity state: boutons are depressing if $p_i \geq 0.5$ and facilitating otherwise.
